# Round the Hospitals

**Published:** 1917-12-08

**Authors:** 


### ROUND THE HOSPITALS.

A LARGE and representative meeting, presided over by Dr. Hope, Medical Officer of Health, was held in the Royal Institution, Liverpool, on Tuesday, November 27, when the aims of the College of Nursing were most ably brought before the meeting by Miss Rundle, Secretary of the College, who urged the nurses to join at once. Miss Gibson then gave a stirring address, warning the nurses against apathy. Mr. Wade Deacon proposed, and Mr. Gaius Coster seconded, a vote of thanks to the chairman and to the speakers. Mr. Wade Deacon said how grieved he had been in reading the nursing papers to see the bitter controversy between the different parties in the nursing world, and expressed the hope that the difference could be settled and unity established. Our columns have contained demonstrable evidence that Sir Arthur Stanley, G.B.E., has shown such extraordinary patience in fighting for unity that, as he deserves, he has won, the sympathy of disinterested people of all sections in support of the College, with the exception of a few agitators who have their own ends to serve, as trained nurses know. The agitators are out to sow disunion, but evidence accumulates that they are being found out by the press, by the public and the workers amongst trained nurses, who are registering with the College in increasing numbers every week. Dr. Hope said there were two things for which the College should be heartily congratulated. He thought one was the success of the Council in obtaining so quickly the ear of the War Office, and subsequently representation of the nursing profession on the Supply of Nurses Committee. The College was also especially fortunate in having such an organisation so well known as the British

**ROUND THE HOSPITALS**—(*continued*).

Women's Hospital Committee in helping to raise the Endowment Fund of the College. Matrons and nurses may recognise Dr. Hope's statement as evidence of the powerful help the College can render trained nurses and their profession in many directions.

Colonel E. M. Bruce Vaughan, whose energy is inspiriting, must have been cheered and heartened by the proceedings at King Edward VII.'s Hospital, Cardiff, when Lady Thomas, who is to be congratulated on her speech and part in the ceremony, presented the Sir William James Thomas Medals for proficiency in nursing as the result of the annual examination. The recipients were: First prize (gold medal), Nurse Edith C. Hooper; second prize (silver medal), Nurse Evelyn F. Tregaskis, who was absent on military nursing duties; third prize (bronze medal), Nurse Hilda C. Evans. The medals were designed by the Welsh artist, Miss Lindsay Williams. King Edward VII.'s Hospital, Cardiff, has done well by establishing a preliminary training-school for incipient probationers on the model of Tredegar House, Bow, and by the appointment of Sister Minet Gould, of the London Hospital, to the important post of sister-tutor in charge of this training-school. The sister-tutor has, by her past training and work, won golden opinions from the authorities of the great hospital from which she comes. She has everything at Anthony House, 30 Newport Road, well arranged and in excellent order, and the Cardiff school should prove widely popular and encouragingly efficient. They do not let the grass grow under their feet at Cardiff, and energy and efficiency are two horses which portend victory and keep a straight course to success.

Miss E. A. Montgomery Wilson, R.R.C., matron of King Edward VII.'s Hospital, Cardiff, has sent us a communication which is likely to arouse criticism. Miss Wilson points out that the demands of the war have created a widespread interest in the nursing profession, and that King Edward VII.'s Hospital is trying to adapt itself to the new requirements. With this in view the training-school for nurses above referred to has been established, where candidates for probationership receive eight weeks' preliminary training before entering the wards of the hospital. A feature of this scheme which is said to have appealed to a number of applicants is the extension of the period of training from three to five years, with payment for every year of training—*i.e.* first year, £10; second, £16; third, £20; fourth, £35; fifth, £40. The term of agreement for nurses will be extended to five years, a three years' certificate being granted on completion of the three years' training, but each nurse is expected to remain in the service of the hospital for a further two years, working upon its private or general staff. We gravely doubt whether this scheme is likely to prove popular with the nurses, who, at the end of their third year, may wish to obtain wider experience and to spend a further two years in following a college course, or studying special branches of their work, or in travel, to fit them to the fullest extent possible for the higher appointments in the nursing profession. We may add that the next term for the preliminary training at Cardiff will commence early in the year, and intending candidates (aged not less than twenty-one years) should apply to Miss Wilson without delay.

Miss Stansfeld's address at the Poor-Law Infirmary Matrons' Association meeting contains several excellent points. The Archbishop of Canterbury's declaration from the pulpit of Westminster Abbey on the day of Their present Majesties' coronation, "I am among you as One that serveth," recalled Their Majesties' personal labours for their people and the good of the Empire. The fact that the King and Queen never spare themselves was properly emphasised, as an example to be followed. To everyone opportunities come to serve their nation and kindred in this war, which has already afforded proof that heroism and patriotism of the noblest are still instinct in the manhood of the Empire. For women, too, the war has improved their position and given them wider powers. In Miss Stansfeld's view it has taught us to lead simpler and less luxurious lives—we wish we could believe that this was true of every family in the land!—to think more of others and less of ourselves, and continuously to realise more clearly and fully our individual responsibilities towards the Empire. The war has defined the position of the Poor-Law nurse, matrons and sisters, and all of them have given proof that in knowledge, skill, and power of devotion to the sick they are second to none.

Again, Miss Stansfeld spoke forcibly and well in regard to work in the limelight near the fighting-line, and in the comparative quietude and obscurity of the Homeland. While some Poor-Law matrons are doing splendid work in the military hospitals, both in England and abroad, many matrons have elected to remain at their posts in Poor-Law infirmaries and to carry through the many difficulties caused by the war, including (*a*) the shortage of sisters, resulting in third-year probationers acting in their place, whose lack of experience entails much more responsibility and watchfulness on the part of the matron; (*b*) the increasing difficulty of getting probationers, the occupations of a V.A.D. having proved more attractive; (*c*) the shortage of male and female domestic staffs, occasioned by the higher wages given in munition factories, difficulties which entail on the matron much more oversight in the preparation of food, its punctual and careful serving, and the enforcement of cleanliness throughout her institution. All matrons in these circumstances labour under heavy stress and strain, and their patriotism is as great as, or greater than, that of matrons who have been able to undertake service in military hospitals and obtain the Royal Red Cross. Miss Stansfeld rightly insists that

**ROUND THE HOSPITALS—(*continued*).**

much of the best work of the world is unseen work, and there is, and must be, many a matron whose work, though of the highest excellence, may never appear before the footlights or be recognised by the world.

---

Miss Stansfeld spoke with genuine feeling and force in dealing with the special, arduous, and often ennobling duties which members of the nursing profession discharge in Poor-Law infirmaries. She welcomed the action of the Council of the Poor-Law Infirmary Matrons' Association in inviting superintendent nurses to join it as Associates, for they have under their care a large number of chronic cases which, day after day, month after month, year after year, require skilled patience and devotion. Let us give a few of her examples. A man lying on a bed in the corner of a sick ward, suffering from rheumatoid arthritis and heart weakness. The patient is an elderly, gentlemanly, refined, bright, cheerful, and devout man, taking his suffering as his cross in life, and simply waiting for his call to rest. Then there are three young men, who have been bedridden for years—one an artist of no mean skill, one a lover of all good books who, as we find him in his bed, is reading "The Roadmender"—and all three are bright, responsive, and remarkable for their patience. Is it possible for any nurse, anywhere, to render higher or more valuable service than in caring for such patients as these, of whom there are many to be met with in Poor-Law work? The patients are not all patient, and there are some unruly and restless ones, who demand on the part of the superintendent nurse the exercise of tactful sympathy, with its calming influence, which may mercifully reconcile them to the knowledge that the bed is their allotted place whilst life lasts. Could any service prove of higher or greater value than work of this kind, done *con amore* by many a superintendent nurse? Miss Stansfeld had a patient thirty years ago in Mark Ward, St. Bartholomew's Hospital, whom she had to nurse. He was a bill-sticker by trade, and, before leaving the hospital, he said to her: "Nurse, I am different to what I was when I entered the ward. When the lights are low at night Sister comes round and talks so quietly to us; she has made a better man of me."

---

Let us remember, too, that a matron's life is, by virtue of her position on the staff, one of peculiar loneliness. The greater the responsibility, the greater the sacrifice. Her life is a dull round of little sacrifices none the less trying because they are small. The tone of a nursing staff is the direct result of the character of the matron. Thirty years' experience as an inspector emboldened Miss Stansfeld to declare: "If you will show me a nursing staff I will tell you something of the character of the matron." Florence Nightingale held: "A good nurse should be the Sermon on the Mount in herself." If this be true in its application to nurses, how much more is it of the matron who is called upon to train and to influence the many over whom she is in authority.

---

Our readers have been greatly interested, no doubt, in the account we gave of the memorial reredos to the late Sister-Matron Katharine Monk, of King's College Hospital. Hers was a great character, and the famous Dr. Butler, Master of Trinity, Cambridge, impressed on his undergraduates that a great character is like a great sanctuary, you visit it and leave it, and then duty begins. Miss Stansfeld properly insists that every matron should be a sanctuary to her nurses. She should be their guide, counsellor, and friend, the one person to whom they can all always go for help and advice. The smooth working of a great hospital depends mainly on the character and apprehension of the matron. One great matron, now out of the Service, told Miss Stansfeld that she always made a point of going to the room of a new probationer on the first night of her arrival. Sitting on her bed, she would have a quiet talk with her, warning her of the dangers of a nurse's life and pointing out its ideals. We know—everybody of great experience in hospital life knows—that the nurses who have had the good fortune to be trained under such a matron reflect something of the personality of a devoted woman of this type, for their character does in fact prove a sanctuary to all who are trained under them and who have come under their influence. We could wish that every matron, especially in this war-time, who is working in the Homeland, would make a point of satisfying herself whether she has any sister Gorgons, one or more, on her nursing staff who, by harsh and inconsiderate treatment of the probationers, may prove every hour she is on duty to be a woman who ought to have nothing whatever to do with their training or discipline, or at all. These are not days in which it should be possible for any sister Gorgon to remain at work in any well-managed and reputable British hospital.

---

Our letter-box has caused us increasing uneasiness of late, owing to the number of complaints of inconsideration, unkindness, and want of sympathy on the part of some sisters and nurses in fever hospitals and elsewhere. At the meeting of the Poor-Law Infirmary Matrons' Association Miss Stansfeld, chief lady inspector of the Local Government Board, justified our anxiety by stating in the course of her speech that not long ago she had found a nurse crying bitterly in her room, who, on being asked the reason, said: "I have lost my sister, and she suffered terribly. The nurse was so unkind when I went into the ward to inquire how my sister was. The nurse said: 'Oh! I think she feels pretty rotten. Sister is not anxious.' Upon going to see my sister she told me that the sister of the ward had told her not to behave like any old woman." Two days after the patient died. We welcome Miss Stansfeld's insistence on the necessity for kindness and sym-

ROUND THE HOSPITALS—(*continued*).

pathy by the nurses towards the friends of the patients as well as to the patients themselves. We hope Poor-Law matrons, and, indeed, all matrons throughout the country, will have an eye to this necessity, which seems to be emphasised under war conditions and the absence of so many members of the permanent staff on other duties.

---

Mrs. Luke Paget, wife of the Bishop of Stepney, speaking of the development of the Poor-Law system, which dates back to the days of Gregory the Great in the Early Christian era, fixed 1601 as the year when the first real Poor-Law system came into operation. In her early days Mrs. Paget had to go daily for the supply of meat for the patients in St. Bartholomew's Hospital. At that time "the ward was a long hall with a chapel at one end and in each bed any number of patients from two to eight." Mrs. Paget was in favour of talks with the nurses—nonsense talks, or talks about books, art, and so forth. This plan would, she felt, help to widen considerably the nurses' outlook, for they required more relief from study and to have the benefit of new ideas brought to them from the outside world. This suggestion was heartily endorsed by Miss Dodds and Miss Dowbiggin, and all three speakers invited lay people of education to visit the infirmaries and the nurses in furtherance of the plan suggested. Miss Seymour Yapp, of Ashton-under-Lyne, was in favour of provincial branches in connection with the Poor-Law Infirmary Matrons' Association, to enable the various members in the provinces to discuss their difficulties and so prepare themselves to take part in the general meetings in London. This suggestion was approved, and is to be followed by the Association. Miss Yapp further suggested that the matrons of the larger infirmaries should call a meeting in their respective districts with representatives of the smaller Unions, and that the scope of the Association should be widened by including head nurses as well as superintendents. Miss Amy Hughes, who has had great experience of nurses trained in Poor-Law infirmaries, has found them as Queen's nurses admirably fitted for nursing the old and chronic patients as well as the acute cases. Other speakers were Miss Smith, of Hendon, and Miss Girdlestone. Miss Stansfeld bore eloquent testimony to the special debt of gratitude which all owed to Miss Barton, their president, who had kept the balance so true. Both Miss Barton, for her untiring services on behalf of the Association, and Miss Alsop, for arranging so many meetings at Kensington Infirmary, were cordially thanked.

---

Col. William Hunter, C.B., M.D., F.R.C.P., has paid a notable tribute to the personality, work, and achievement of the late Dr. Elsie Inglis, the originator of the Scottish Women's Hospitals, who died last week after landing in England from Russia. In the early days of the war Miss Inglis, who for many years had held the post of joint surgeon to the Edinburgh Hospital and Dispensary for Women and Children, conceived the idea of forming the Scottish Women's Hospital, staffed entirely by women. In those days the War Office refused to consider a hospital so staffed, and Dr. Inglis and the Scottish Women's Hospital Committee placed themselves at the disposal of our Allies, by whom they were accepted. The work they have done since that time is well known, and of Dr. Inglis Col. Hunter writes in the *Times* of November 29: "It was my privilege and happiness to see much of her work in Serbia early in 1915. . . . I have never met with anyone who gave me so deep an impression of single-mindedness, gentle-heartedness, clear and purposeful position. I regard her death as a grievous loss to noble philanthropic effort, and her memory as one to be proud of and treasured by the nation she so nobly represented, as it will for all time be treasured by the Serbian nation, whom she so nobly and successfully served." Her Majesty the Queen has sent a message of sympathy to the late Dr. Inglis' sister, and an appreciation of the splendid services rendered by her to Serbia, which can never be forgotten. The funeral service in St. Giles' Cathedral, Edinburgh, was attended by a large number of mourners, amongst them being the Serbian Minister, representing the King and the Prince Regent of Serbia. Dr. Wallace Williamson's address at the service in the cathedral was a stirring tribute to one whose name, he averred, would be for ever linked with the great souls who had led the van in womanly service for God and man.

---

"Ierne," our Irish Correspondent, writes: "Your able and trenchant exposure of hypocritical protest against raising a National Endowment or Benevolent Fund for the benefit of the profession is much appreciated in nursing circles here, excluding, of course, Mrs. Fenwick's satellites. Has it been forgotten that the training of nurses found its origin in the national thank-offering of £50,000 to Florence Nightingale after the Crimean War? The nation owes more than it can ever repay to British nurses for their services in the conflict which is now raging, and if another thank-offering is made to them when the war is over there is no reason to suppose that it will tend to their degradation any more than did the Florence Nightingale memorial."

---

☞ It is our invariable practice to pay for all items of news which we may publish. The minimum payment is 5s. Paragraphs of about twenty lines in length—that is to say, of 150 words or less—are preferred. Contributions should be addressed to The Editor, The Hospital, 28 and 29 Southampton Street, Strand, London, W.C. 2, and, to be in time for the current week's issue, should reach him by the first post on Mondays.

